# A tornado in the family: fetal alcohol spectrum disorder and aggression during childhood and adolescence: a scoping review

**DOI:** 10.3389/fnins.2023.1176695

**Published:** 2023-06-21

**Authors:** Maude Champagne, Jeffrey McCrossin, Jacqueline Pei, James N. Reynolds

**Affiliations:** ^1^Centre for Neuroscience Studies, Queen’s University, Kingston, ON, Canada; ^2^School of Social Work, McGill University, Montreal, PQ, Canada; ^3^Department of Educational Psychology, University of Alberta, Edmonton, AB, Canada

**Keywords:** aggression, intervention, fetal alcohol spectrum disorder (FASD), scoping review, child, adolescent

## Abstract

**Background:**

Aggression exhibited by children and youth with Fetal Alcohol Spectrum Disorder (FASD) toward family members is a major cause of stress and anxiety for caregivers, but relatively little attention has been directed toward designing interventions specific to this phenomenon. In light of the serious negative impact of this issue for families, a scoping review was undertaken to summarize the evidence available on psychosocial interventions that may mitigate the frequency and severity of aggression exhibited by children and youth with FASD toward family members.

**Methods:**

This review was designed using PRISMA-SCR and JBI scoping review guidelines. Three databases were searched in August 2021: EMBASE, PsychINFO, and Medline.

**Results:**

A total of 1,061 studies were imported for screening with only five studies meeting full eligibility criteria. None of the interventions were aimed at specifically targeting aggression and instead reported on broader constructs of externalizing behaviors such as hyperactivity. The interventions were limited to school-aged children. Studies reported primarily on child outcomes while only one reported on family related outcomes.

**Conclusion:**

Following from this review of the literature, we argue that aggression is a related but separate construct from other behavioral problems most frequently targeted by parenting interventions. Given the often dire consequence of aggression displayed by children and youth with FASD and the limited number of studies, there is an urgent need for research on how to support families to manage this specific type of behavior in this population.

## 1. Introduction

Fetal Alcohol Spectrum Disorder (FASD) encompasses a range of neurodevelopmental outcomes that may occur as a consequence of prenatal alcohol exposure (PAE). A recent prevalence study estimated that the rate of FASD in the general population of Canada is approximately 4% (Popova et al., 2019). FASD is associated with deficits in multiple behavioral domains, including executive and adaptive functioning skills, emotional regulation, and learning and communication (Cook et al., 2016). Families raising children and youth with FASD report experiencing high levels of stress specifically in relation to dealing with maladaptive behaviors (Watson et al., 2013) and caregivers have reported that childhood aggression, violence and temper tantrums are the most difficult challenges for families to manage (Green et al., 2014). In a meta-analysis, Tsang et al. (2016) found aggression to be among the most reported concerns of parents and teachers of children with FASD, and that children with FASD exhibited higher levels of aggressive behavior compared to children diagnosed with attention-deficit/hyperactivity disorder (ADHD) without PAE (Tsang et al., 2016). Families experiencing aggression from their child with FASD in adoptive homes have reported placement instability—a situation aggravated by the COVID-19 pandemic (Champagne et al., 2023).

A recent report from the National Consortium on Aggression toward Family/Caregivers in Childhood and Adolescence, that included interviews with families raising children and youth with FASD in Canada, highlighted this issue as an urgent matter (AFCCA, 2021). Child-related impacts that were reported included exclusion from school and community settings, damaged relationships, adverse mental health outcomes and diminished self-esteem. The impacts reported for caregivers and siblings encompassed a range of issues from physical injury to psychological trauma, financial strain, and negative mental health. Furthermore, families experiencing aggression reported receiving very little support to assist them and their child which led, in some instances, to involvement with the justice system or the child being removed from the home. These difficult situations were compared to a tornado striking the family, leaving damage and devastation behind; a storm needing to be weathered with support and understanding from those around. That is, caregivers need to be supported in their role to provide a safe environment for the affected child and for the whole family (AFCCA, 2021). If the appropriate support is provided, the evidence suggests that children and youth with FASD can thrive, live safely and enjoy a good quality of life (Pei et al., 2019). The strengths of youth with FASD have been reported in several studies based on a recent review such as their kindness, loyalty, forgiveness, and many creative talents (Flannigan et al., 2021).

Recently, Canadian researchers completed a synthesizing review of aggressive behavior and violence in the context of FASD (Joseph et al., 2022). The authors argue that understanding the mechanisms underlying the behavioral presentation of a child/youth with FASD requires a consideration of the convergence of genetic, environmental and neurophysiological developmental factors. In particular, the authors point to the importance of the interaction between prenatal alcohol exposure and the postnatal environment, especially exposure to trauma, violence and other adverse childhood experiences, as key factors driving the occurrence of aggression in children/youth with FASD (Joseph et al., 2022). To further our understanding of aggression in FASD, this scoping review was developed to identify how aggression is addressed in the context of psychosocial interventions for children and adolescents living with FASD to promote healthy outcomes.

Aggression is defined as the delivery of any form of definite and observable harm-giving behavior toward any target (Ramirez and Andreu, 2006). Although most often aggression in children is thought of as physical in nature (e.g., hitting, kicking, biting, or throwing objects) other types of aggression include psychological (e.g., shaming, threatening) and relational aggression (e.g., malicious gossiping). Aggression can be reactive or proactive in nature (Kempes et al., 2005). Reactive aggression refers to the combative reaction to a perceived threat, while proactive aggression refers to behavior in expectation of achieving some goal, such as domination or control (Kempes et al., 2005). This dichotomous model of reactive-proactive aggression has been critiqued for oversimplifying a complex issue (Bushman and Anderson, 2001). Additional subtypes of aggression have been defined in the psychiatry literature to better understand the phenomenon and plan treatment accordingly (Connor et al., 2019). For example, impulsive aggression (IA) is a reactive and maladaptive subtype of aggression that usually is expressed in individuals with an impaired central nervous system (Waltes et al., 2016). IA is a common form of aggression reported in relation to several neurodevelopmental disorders such as autism spectrum disorder (ASD) and ADHD, as well as traumatic brain injury and mental health disorders (Connor et al., 2019). Given the applicability in related neurodevelopmental disorders, IA may be of interest in the context of FASD as well. IA may be identified by common characteristics that describe the unplanned nature of the aggression, challenges in regaining composure, and demonstration of remorse: “Characteristics include sudden, intense aggression inappropriately expressed in relationship to environmental precipitants. The individual may have frequent aggressive episodes, difficulty terminating aggression, and remorse when the episode ends” (Connor et al., 2019).

Aggression during childhood and adolescence occurring in the general population has several proposed etiologies that have been described in the scientific literature based on interactional models. The model describing IA suggests that central nervous system dysfunction interacts with other factors such as social adversity and genetics (Connor et al., 2019). Little is currently known about possible etiological factors of aggression specifically in the FASD population but research on other neurodevelopmental disorders, such as autism spectrum disorder (ASD), found that risk factors included greater impairment in cognition, language, and adaptive behavior (Politte et al., 2019). Nevertheless, it has yet to be determined which factors could play a role in the issue of aggression for the FASD population. Understanding the etiology of aggression in FASD is important to plan the proper course of intervention to improve the quality of life of these children and their caregivers.

Aggression is not well understood in children and youth with FASD, and there are no published reviews of psychosocial interventions addressing aggression in this population. Given the emotional and physical consequences of aggression on the quality of life of children and their caregivers, it is imperative to address this issue. For these reasons, a scoping review methodology was selected to explore the interventions for the issue of aggression in children and youth with FASD, map the current evidence, better understand the key concepts, and identify knowledge gaps related to this topic.

The initial aim of this scoping review was to assess the evidence available on psychosocial interventions addressing the impact of aggression by children and youth with FASD toward family members. However, the initial search of the literature did not yield data specific to the family context. Therefore, we adapted the review to instead summarize more broadly the evidence available on psychosocial interventions for aggression displayed by children and youth with FASD or Prenatal Alcohol Exposure (PAE) as well as the gaps in knowledge. We formulated the following research questions: (1) What are the terms most used in the intervention literature to describe aggression displayed by children and youth with FASD or PAE? (2) What are the current psychosocial interventions reported in the literature that are used to respond to this issue? (3) What are the current gaps and limitations in the literature regarding psychosocial intervention for aggression displayed by this population? This study is a preliminary step in developing a richer conceptualization for aggression in the context of children and youth living with FASD or PAE.

## 2. Methods

### 2.1. Design

This scoping review was designed using the PRISMA-SCR (Tricco et al., 2018) and JBI scoping review guidelines (Peters et al., 2015).

### 2.2. Search strategy

Databases were determined in collaboration with a professional librarian at Queen’s University and a search protocol was designed for each database. Key search terms were determined by three researchers in the field of FASD (James Reynolds, Jacqueline Pei, and Maude Champagne) and informed through an iterative process in the early stages of preparing for this review. Terms related to FASD, PAE, and aggression, violence or disruptive behaviors were used to identify articles that may contain data related to the research questions. The search process took place in August 2021 and included Embase, Medline and PsychINFO databases.

### 2.3. Article screening

**FIGURE 1:**
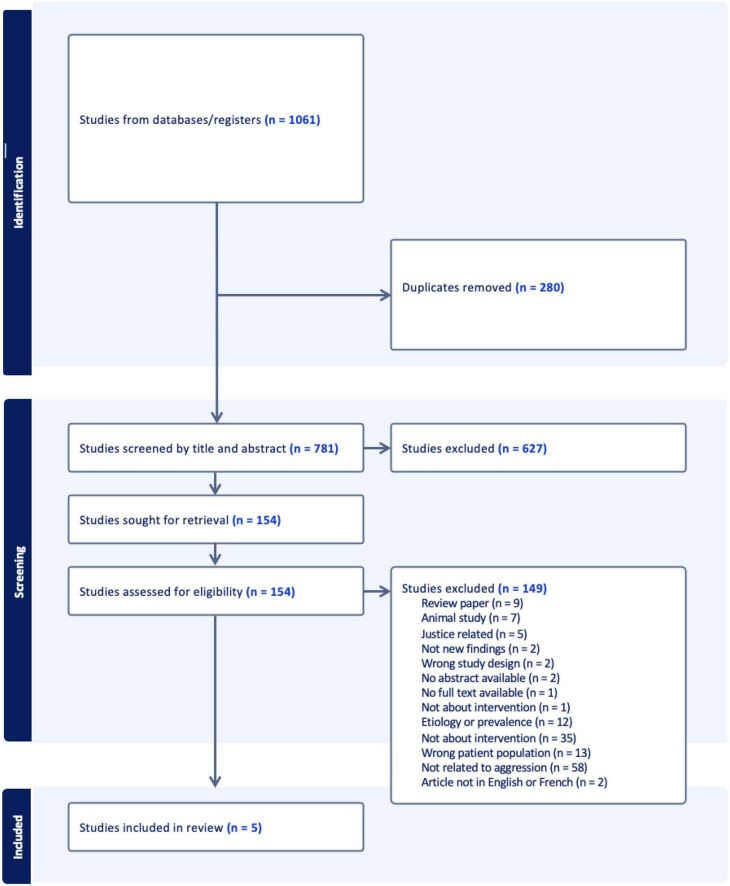
PRISMA flow diagram.

The PRISMA- Scoping Review Extension was used to guide the screening process (Tricco et al., 2018). Search results were exported to the systematic review software Covidence^1^ and duplicates were removed. In the first stage of screening, the first author screened titles and abstracts to remove any articles unrelated to psychosocial issues in FASD. During the second stage of screening, the first and second authors identified the inclusion and exclusion criteria through an iterative process of team discussion as authors became more familiar with the available literature. To be included in our review, articles needed to report on the evaluation of a psychosocial intervention targeting aggression or other externalizing behavior problems in children or youth with FASD or PAE under the age of 18. We placed no restrictions on study design, and as such, we included qualitative, quantitative, and mixed-methods studies. Review papers, book chapters, and gray literature were excluded. Only articles published in English or French were included. No restrictions were placed on the date of publication. The first and second authors conducted a third screening phase, reviewing the full-text of articles to assess eligibility based on the inclusion and exclusion criteria. Consensus was reached on all disagreements through team discussion. A PRISMA flow diagram is provided (see Figure 1).

### 2.4. Data extraction and analysis

A data extraction tool was created by the first author based on the research questions and the JBI Scoping Review guidelines (Peters et al., 2015). The tool included study characteristics, measurement tools, parental involvement in the intervention, and key findings. The tool was tested and modified through an iterative process by the first and second authors using three articles. Data from all five articles included in the review were extracted using the final extraction tool. Data extracted from two articles that used the same dataset were merged (Petrenko et al., 2017, 2019).

## 3. Results

The initial search resulted in 1,061 studies from which 280 duplicates were removed (Figure 1). Subsequently, 627 studies were removed from the remaining 781 studies during the initial screening process by the first author. The full texts of the remaining 154 articles were screened for eligibility. Approximately one third of the studies screened at this stage were not related to aggression (*n* = 58), and several were not related to psychosocial interventions but focused instead on etiology (e.g., brain imaging or medication), adults with FASD, or were review articles. A total of five studies met full eligibility criteria and were included in the data extraction phase of the review.

### 3.1. Intervention characteristics

Four interventions were identified in this review for children and youth with FASD (Table 1). The *GoFAR*, *Families on Track*, and *Alert Program* interventions all aimed to improve child behaviors by targeting either executive functioning or emotional regulation (Coles et al., 2015; Nash et al., 2015; Petrenko et al., 2017). The *Math Interactive Learning Experience* (MILE) intervention program aims to improve math performance and behaviors (Coles et al., 2009). None of the interventions described in the articles were designed specifically to modify patterns of aggression. Similarly, none of the studies measured aggression as a construct. Instead, each study reported on other behavior-related constructs, such as disruptive behaviors, externalized behaviors, emotion regulation, temper tantrums, frustration tolerance, impulsivity, and destructiveness.

**TABLE 1:** Intervention characteristics.

References	Intervention description	Target of intervention parent involvement in length of intervention	Parent involvement in intervention	Length of intervention
Coles et al., 2009	MILE math intervention, caregiver education, if needed case management, social work or psychiatric services were provided.	Evaluating the persistence of the effect of an intervention on math performance and behaviors.	Parents were given workshops and weekly assignments	6 weeks of math intervention and two parent workshops
Coles et al., 2015	The first element of the intervention included a psychoeducational workshop for parents on the neurobehavioral implications of prenatal alcohol exposure. The second element lasted for 5 weeks and include the GoFAR videogame for children where they learn a metacognitive strategy to focus and plan, act, and reflect; and a simultaneous parent training program on supporting children to develop behavioral regulation skills. The third element of the intervention involved 5 weekly behavior analog therapy sessions where parent-child dyads learn to implement the metacognitive strategy in the context of problem behavior observed at home.	To test the impact of a metacognitive, video game based intervention, parent psychoeducation and training on emotion regulation and disruptive behaviors.	Parents were involved in dyadic therapy sessions and individual psychoeducational workshops	15 sessions: 5 GoFAR for the children 5 individual sessions with the parent, 5 dyadic session
Nash et al., 2015	Alert Program for Self-Regulation targets improvement in self-regulation through sensory and cognitive processing. The therapy involves three successive stages organized around a car analogy of car engine speeds representing recognition and modulation of sensory and cognitive processing.	To evaluate the impact of the Alert intervention on aspects of executive functioning in children with FASD such as behaviors and social skills.	one parent session	12 sessions of therapy with the child
Petrenko et al., 2017	Families on Track: This intervention involved a neuropsychological and diagnostic evaluation and two treatment components. The caregiver treatment involved 1.5 h in-home sessions with a facilitator reviewing skills taught in the child group sessions to encourage generalization of skills to the home environment. The child treatment involved a small group (6–10 children) of which a portion (3–4) had diagnoses of FASD while the remainder were typically developing children in the target age range. The child group sessions targeted social and emotional skill development.	To pilot an intervention program targeting social and emotional development in children with both a child focused intervention and concurrent parent-focused intervention. The program targets key risk factors and protective factor.	Parents received training to support generalization of child skills in the home environment	30 weeks long

The MILE program consists of 6 weeks of math interventions and two parent workshops (Coles et al., 2009). Families were also provided with behavior problem case management, social work, psychiatric consultations, and strategies to foster child self-regulation skills.

The *GoFAR* videogame teaches children metacognitive strategies to focus, plan, act and reflect (Coles et al., 2015). This program has several caregiver-focused elements such as psychoeducation training and dyadic sessions. *GoFAR* consists of 15 sessions; 5 sessions for the children, 5 individual sessions with the parent and 5 dyadic sessions.

The *Alert Program* is child-focused and uses a car analogy to teach the child to use strategies for modulation of sensory and cognitive processing (Nash et al., 2015). The impact of the program is tested on the child’s executive functioning skills, behaviors and social skills. The program consisted of 12 sessions with the child and 1 parent workshop.

*Families on Track* has two published manuscripts (pilot and 6 months follow-up) assessing the impact of the intervention on the emotional and social development of the child (Petrenko et al., 2017, 2019). The 30-week program described by Petrenko et al. (2017) centered on preventing secondary conditions and improving family functioning, rather than improvement of child behavior problems. FASD diagnostic services were offered. Children received group therapy and caregivers received training to help the child generalize the skills at home.

### 3.2. Study characteristics

Out of the five articles eligible for this scoping review, four were from the USA and one was from Canada (Table 2). Studies were published between 2009 and 2019 with four published in the past 6 years. All were randomized control trials conducted in universities or pediatric clinics using quantitative methodologies. All studies had relatively small sample sizes between 25 and 54 and two of the studies were pilots.

**TABLE 2:** Study characteristics.

	References	Country	Context	Study design	Sample size
1	Coles et al., 2009	United States	Pediatric neurodevelopmental exposure clinic	RCT^1^	54
2	Coles et al., 2015	United States	Pediatric neurodevelopmental exposure clinic	3-arm RCT	30
3	Nash et al., 2015	Canada	Pediatric hospital or accredited FASD diagnostic clinic	RCT	25
4	Petrenko et al., 2017	United States	University clinic	RCT	30

^1^Randomized control trial.

### 3.3. Sample characteristics

Participating children were all diagnosed with FASD (Canadian guidelines) (Cook et al., 2016), FAS or pFAS (United States guidelines) (Astley, 2013) (Table 3). The age range of the children or youth was between 5 and 12 years, and studies included predominantly children living with foster, adoptive or kinship carers. Three studies included child race, two of which reported that the majority of participants were of Caucasian descent.

**TABLE 3:** Sample characteristics.

	Coles et al. (2009)	Coles et al. (2015)	Nash et al. (2015)	Petrenko et al. (2017)
Age of child	Range: 5–10 years old Mean (SD): 6.5 (2.0)	Range: 5–10 years old Mean (SD): 7.5 (1.4)	Range: 8–12 years old Mean (SD): 10.3 (1.7)	Range: 4–8 years old Mean (SD): 6.52 (1.31)
Relationship to caregivers	71.4% adopted by non-relatives	100% legal guardianship or adoptive caregiver	Adopted: 75% Foster: 17% Kinship: 8%	Adopted or foster: 81.3% Kinship: 18.8%
Age of caregiver	Mean (SD): 43.9 (7.2)	Not reported	Not reported	Mean (SD):45.77 (8.97)
Race and culture	64% white (intervention group)	40% Caucasian 40% Mixed -raced 10% Black	Not reported	87.5% white
Gross household income or socioeconomic status	Range of household income: $35,000-$49,000	Not reported	Socio-economic status: Low:42% Medium: 42% High: 16%	Family income: mean (SD) $90,312$ ($49,378$)

### 3.4. Outcomes and tools

Child behaviors were assessed using the Child Behavior Checklist (CBCL) (*n* = 2), Eyberg Child Behavior Inventory, Emotion Regulation Checklist, Impairment Rating Scale, Disruptive Behavior Record Form, or Behavior Rating Inventory of Executive Function (BRIEF) (Table 4). Only one study included parental outcomes measures (Petrenko et al., 2017).

**TABLE 4:** Outcomes measures and findings.

References	Descriptive words for aggression	Child measures of behavior	Key findings for constructs related to aggression	Parent outcome measures
Coles et al., 2009	Externalized behavior problems	CBCL^2^ Teacher report form	Aggressive behaviors continued to decline over time and showed a significant difference between Post-tests 1 and 2. For the teacher which had a smaller sample, the summary showed significant differences in externalized behavior	Not reported
Coles et al., 2015	Disruptive behavior, temper tantrums, frustration tolerance, impulsivity destructiveness, aggression,	Disruptive behavior record form	The intervention groups appeared to have improved scores on the disruptive behavior aggregate score with a medium to large effect. However, the results are limited by the small sample size.	Not reported
Nash et al., 2015	Externalizing behavior problems as measured by the CBCL externalizing behavior problems scale and BRIEF behavioral regulation scale.	CBCL BRIEF^3^	At 12 weeks follow-up parents were reporting significant improvement in inhibitory control and social cognition. Reduction in externalized behaviors, and improvements in behavioral and emotional regulation was reported. Behavioral improvements were maintained at 6 months follow-up.	Not reported
Petrenko et al., 2017	Disruptive behavior	Eyberg Child Behavior Inventory Emotion regulation checklist Impairment rating scale	Medium to large size improvement in emotion regulation, significant group difference. A moderate decrease in behavior intensity for the treatment group was noted from pre to post-test, which was maintained at follow-up. No difference between treatment and comparison groups were noted when considering change from pre-test to follow-up. No specific mention of aggressive behavior.	Measures of Caregiver Functioning Knowledge and Advocacy Parenting Practices Interviews Family Needs Met Parenting Sense of Competence Parenting Stress Index Perceived Social Support Scale Self-care Parent Evaluation Inventory Intervention Satisfaction
Petrenko et al., 2019	Same as Petrenko et al. (2017)	Same as Petrenko et al. (2017)	Follow-up data was available for 24 out of the 30 families and showed continued gained for the intervention group in parent self-efficacy and parent knowledge of FASD compared to the comparison group. Decrease in disruptive behaviors in both groups although improvement in child self-esteem and emotional regulation were diminished.	Measures of Caregiver Functioning Knowledge and Advocacy Family Needs Met Parenting Stress Index
Petrenko et al., 2019	*idem*	*idem*	Follow-up data was available for 24 out of the 30 families and showed continued gained for the intervention group in parent self-efficacy and parent knowledge of FASD compared to the comparison group. Decrease in disruptive behaviors in both groups although improvement in child self-esteem and emotional regulation were diminished.	Measures of Caregiver Functioning Knowledge and Advocacy Family Needs Met Parenting Stress Index

^2^Child behavior checklist (CBCL). ^3^Behavior rating inventory of executive function.

### 3.5. Key findings for aggression-related construct

The MILE intervention study reported a decline in aggressive behaviors over time that achieved statistical significance between Post-tests 1 and 2 (Table 4). Teacher reports also showed significant improvements in externalized behaviors over time (Coles et al., 2009). In the *GoFAR* intervention, the intervention group exhibited improved scores on the disruptive behavior aggregate score with a medium to large effect size (Coles et al., 2015). In the *Alert Program* for self-regulation, parents reported significant improvements in inhibitory control and social cognition at the 12-week followup, accompanied by a reduction in externalized behaviors and improvements in behavioral and emotional regulation. At the 6-month follow-up assessment these behavioral improvements were maintained (Nash et al., 2015). The *Families on Track Program* showed an improvement in emotion regulation with medium to large effect size and a statistically significant group difference. A moderate decrease in behavior intensity for the treatment group was noted from pre- to post-test, which was maintained at follow-up. Follow-up data was available for 24 out of the 30 families and showed continued gains for the intervention group in parent self-efficacy and parent knowledge of FASD compared to the control group. There was a decrease in disruptive behaviors in the control and intervention groups although improvement in child self-esteem and emotional regulation were diminished (Petrenko et al., 2017, 2019).

## 4. Discussion

The limited number of articles eligible for inclusion in this review demonstrates that psychosocial interventions specifically targeting aggression in children and youth with FASD have not been on the research agenda serving this community. Very few psychosocial interventions exist for behavioral challenges in this population, let alone specific to aggression. This despite the fact that families identify aggression and externalizing behaviors as a major concern (Green et al., 2014; Tsang et al., 2016; Champagne et al., 2023). It is difficult to know the prevalence and the extent of the problem with any certainty as this is an issue that often goes unreported due to the stigma associated with aggression in children. This is a field that has not yet been adequately explored. However, from the evidence of positive changes in behavior, cognition, and self-regulation reported in the interventions of this review, it is reasonable to suggest that providing psychosocial interventions targeting aggression in children and youth with FASD is a worthwhile investment that will benefit these individuals and their families.

### 4.1. Family outcomes vs. child outcomes

Our review reveals the relative paucity of evidence for family outcomes in intervention studies targeting aggression and related constructs (e.g., externalized behaviors, disruptive behaviors) in children and youth with FASD. This is a significant knowledge gap given the impact of aggression toward caregivers during childhood and adolescence (AFCCA, 2021). While 3 out of the 4 studies examined in this review included caregivers in their intervention, only one study provided data on caregiver and family outcomes (Petrenko et al., 2017). More recent developments in caregiver training for children with neurodevelopmental disabilities have brought an increased focus on caregiver wellbeing and on the family as a whole (Sikora et al., 2013; Factor et al., 2019). These advances in research priorities acknowledge the importance of caregiver wellbeing on their efficacy in providing care, and the value of the family as an important context in which the child develops (Dykens, 2015). Family cohesion was also a factor identified to promote healthy outcomes in individuals with FASD (Pei et al., 2019).

Including family input and outcomes in intervention studies can provide rich data on family resilience processes that involve risk and protective factors at the level of the child, caregiver, and family (Gunty, 2020). Such data can help improve the design of interventions by identifying pathways through which families thrive in the face of adversity without being limited to child-level factors. Given that impulsive aggression likely stems from impaired brain function compounded by other environmental risk factors, management of this issue should be personalized and multifactorial (Connor et al., 2019). Understanding contributing factors to aggression and its management within the family system could help tailor the treatment by leveraging a broader set of resources beyond interventions that focus solely on child input. Moreover, outcomes should also be measured at the family level rather than solely on the child’s outcome given the importance of caregiver wellbeing and family cohesiveness as mediators of positive outcomes.

There are multiple approaches to assessing family level outcomes available to researchers to measure the impact of interventions on families, including impact of the child’s disability on the family (Trute and Hiebert-Murphy, 2002), family functioning (Epstein et al., 1983; Olson, 2011) quality of life (Hoffman et al., 2006), self-efficacy (Hohlfeld et al., 2018), and resilience (Duncan et al., 2021). Several reviews are available that describe additional family level outcomes and measures for families of children with neurodevelopmental disabilities (Alderfer et al., 2008; Bogossian et al., 2012; Ketelaar et al., 2017) that are highly relevant to the context of children and youth with FASD and aggressive behaviors.

### 4.2. Increasing accessibility and diversity

The studies included in this review were limited in terms of accessibility (i.e., context of delivery) of the interventions and lack of diversity among participants. All studies included in the current review were conducted in-person by clinicians in universities or pediatric centers. Future research should prioritize identifying effective interventions that can be more widely accessible, including consideration of issues such as flexible scheduling according to family availability, location of intervention delivery (e.g., in-person or online), as well as minimizing costs to the family. For example, both GoFAR (do2learn.org) and a subsequent version of Families on Track (Families Moving Forward) (Petrenko et al., 2017, 2019) have been modified for remote/virtual delivery. However, the potential impact of these online interventions for mitigating aggressive behaviors in children and youth with FASD has not been established. The COVID-19 pandemic caused many services to be moved from in-person to online delivery, which created an opportunity for new innovations in intervention delivery methods. Even prior to the pandemic, online parent training interventions were available for children with disruptive behavior (Olthuis et al., 2018), ADHD (DuPaul et al., 2018), and conduct problems (Sanders et al., 2012). Such interventions have many advantages, including offering increased flexibility for caregivers to attend groups at a distance or complete modules on their own time at a fraction of the cost (Olthuis et al., 2018). These programs are, however, exclusively caregiver focused.

Accessibility should also be considered in the context of diversity. The demographic data available for this review were fairly limited but showed a lack of diversity among participants. Most studies included adoptive or foster families, primarily Caucasian and school-age children. Intervention planning should also consider ethnic and racial minorities, Indigenous families, and gender diversity. Biological families are also less inclined to access services due to the stigma attached to the etiology of FASD (Bell et al., 2016). Anti-oppression frameworks could be explored when planning research interventions to ensure accessibility to these groups. Corneau and Stergiopoulos (2012) describe strategies to promote anti-oppression practices in social services such as using self-reflexivity, empowerment and alliance building (Corneau and Stergiopoulos, 2012). Further psychosocial intervention research in FASD for a diverse population is needed.

### 4.3. Future directions

The aim of this scoping review was to map the evidence for psychosocial interventions related to aggression and is a preliminary step in developing a richer conceptualization for aggression in the context of children and youth living with FASD. Many questions remain regarding the prevalence of the issue, the impacts on the children and their caregivers as well as the mechanism of aggression in this population. Meanwhile, interventions specific to the issue of child-to-parent aggression have been developed for other clinical populations (Toole-Anstey et al., 2021). Research is needed to test the efficacy of some of these programs in the FASD population.

Caregivers raising children and youth with FASD who display aggression are facing a highly stressful situation that frequently engenders feelings of isolation and helplessness (AFCCA, 2021; Champagne et al., 2023). The National Consortium on Aggression toward Family/Caregivers in Childhood and Adolescence (AFCCA) made several systemic recommendations (AFCCA, 2021). Among them was the importance of raising awareness about AFCCA in the general population and providing education to professionals and first responders who are often managing these crises when they arise. Educating medical and social services professionals on aggression in this population is also crucial to creating safety for families when accessing support. Furthermore, building a network of peer support for family members and the youth themselves was identified by participants. Finally, creating a system of support for all family members in facing and recovering from the “tornado” that may have hit their family may avoid the entrenchment of these children and youth in the child welfare and youth justice systems. Recommendations were also made to develop multisectoral and personalized interventions that are strength-based and non-stigmatizing as well as delivered in a timely manner. Consulting with caregivers and individuals who have lived-with experience is essential for guidance on ways forward.

### 4.4. Strengths and limitations

A strength of the current review is the collaboration with two national experts in the field of FASD, one clinician-scientist and a professional librarian who advised the search criteria. A limitation of this review is the lack of the term “aggression” in our search. However, most researchers use other terms to describe the problematic behaviors they are aiming at targeting with their intervention. Scoping reviews are also very broad, and it is possible that the search strategy employed may have missed interventions that could provide additional insight into the conceptualization of aggression in FASD. Interventions aimed at other problems commonly associated with FASD such as sleep disturbance, sensory integration disorder, and developmental trauma may also reveal potentially interesting clues to the problem of aggression. For instance, sleep disturbance is associated with increased externalizing behavior problems, thus sleep intervention could possibly be part of the solution for some individuals.

## 5. Conclusion

While caregivers have described aggression as an area of great concern to help them care for their children and youth with FASD, no intervention has been designed to specifically target outcomes related to this construct. The very limited number of interventions identified in this review were all aimed at changing the child’s behavior, improving self-regulation, and other cognitive skills. More research is needed on the etiology of aggression in this population to advance the development of evidence-based interventions.

## Author contributions

MC, JM, and JP developed the search strategy. MC conducted the search and screening of articles retrieved, conducted the analysis, and drafted the first version of the manuscript. All authors contributed to the conceptualization of this scoping review and reviewed drafts of the manuscript.

## Footnotes

1
https://www.covidence.org/

## References

[B1] AFCCA (2021). *National consortium on aggression toward family/caregivers in childhood & adolescence (AFCCA).* AFCCA: Washington, MD.

[B2] Alderfer M. A. Fiese B. H. Gold J. I. Cutuli J. J. Holmbeck G. N. Goldbeck L. (2008). Evidence-based assessment in pediatric psychology: family measures. *J. Pediatr. Psychol.* 33 1046–1061. 10.1093/jpepsy/jsm083 17905801 PMC2639492

[B3] Astley S. (2013). Validation of the fetal alcohol spectrum disorder (FASD) 4-digit diagnostic code. *J. Population Ther. Clin. Pharmacol.* 20 e416–e467. 24323701

[B4] Bell E. Andrew G. Di Pietro N. Chudley A. E. Reynolds J. Racine E. (2016). It’s a shame! Stigma against fetal alcohol spectrum disorder: examining the ethical implications for public health practices and policies. *Public Health Ethics* 9 65–77. 10.1093/phe/phv012

[B5] Bogossian A. Lach L. Saini M. (2012). “Environmental factors: support and relationships,” in *Measures for children with developmental disability: an ICF-CY approach*, ed. Majnemer A. (London: Mac Keith Press), E310–E399.

[B6] Bushman B. J. Anderson C. A. (2001). Is it time to pull the plug on hostile versus instrumental aggression dichotomy? *Psychol. Rev.* 108 273–279. 10.1037/0033-295x.108.1.273 11212630

[B7] Champagne M. Willis R. Reynolds J. (2023). COVID-19 pandemic challenges for families of children and youth impacted by fetal alcohol spectrum disorder. *J. Dev. Disabil*. 28.

[B8] Coles C. D. Kable J. A. Taddeo E. (2009). Math performance and behavior problems in children affected by prenatal alcohol exposure: intervention and follow-up. *J. Dev. Behav. Pediatr.* 30 7–15. 10.1097/DBP.0b013e3181966780 19194327

[B9] Coles C. D. Kable J. A. Taddeo E. Strickland D. C. (2015). A metacognitive strategy for reducing disruptive behavior in children with fetal alcohol spectrum disorders: GoFAR pilot. *Alcohol. Clin. Exp. Res.* 39 2224–2233. 10.1111/acer.12885 26503069

[B10] Connor D. F. Newborn J. H. Saylor B. H. Lawrence Scahill A. S. Robb A. Jensen P. (2019). Maladaptive aggression: with a focus on impulsive aggression in children and adolescents. *J. Child Adolesc. Psychopharmacol.* 29 576–591. 31453715 10.1089/cap.2019.0039 PMC6786344

[B11] Cook J. L. Green C. R. Lilley C. M. Anderson S. M. Baldwin M. E. Chudley A. E. (2016). Fetal alcohol spectrum disorder: a guideline for diagnosis across the lifespan. *Cana. Med. Assoc. J.* 188 191–197. 10.1503/cmaj.141593 26668194 PMC4754181

[B12] Corneau S. Stergiopoulos V. (2012). More than being against it: anti-racism and anti-oppression in mental health services. *Trans. Psychiatry* 49 261–282. 10.1177/1363461512441594 22508637

[B13] Duncan J. M. Garrison M. E. Killian T. S. (2021). Measuring family resilience: evaluating the walsh family resilience questionnaire. *Fam. J.* 29 80–85. 10.1177/1066480720956641

[B14] DuPaul G. J. Kern L. Belk G. Custer B. Daffner M. Hatfield A. (2018). Face-to-face versus online behavioral parent training for young children at risk for ADHD: treatment engagement and outcomes. *J. Clinical Child Adoles. Psychol.* 47 (Suppl. 1), S369–S383. 10.1080/15374416.2017.1342544 28715272

[B15] Dykens E. M. (2015). Family adjustment and interventions in neurodevelopmental disorders. *Curr. Opin. Psychiatry* 28 121–126. 10.1097/YCO.0000000000000129 25594421 PMC5348480

[B16] Epstein N. B. Baldwin L. M. Bishop D. S. (1983). The mcmaster family assessment device. *J. Mar. Fam. Ther.* 9 171–180. 10.1111/j.1752-0606.1983.tb01497.x

[B17] Factor R. S. Ollendick T. H. Cooper L. D. Dunsmore J. C. Rea H. M. Scarpa A. (2019). All in the family: a systematic review of the effect of caregiver-administered Autism Spectrum Disorder interventions on family functioning and relationships. *Clin. Child Fam. Psychol. Rev.* 22 433–457. 10.1007/s10567-019-00297-x 31363949

[B18] Flannigan K. Wrath A. Ritter C. McLachlan K. Harding K. D. Campbell A. (2021). Balancing the story of fetal alcohol spectrum disorder: a narrative review of the literature on strengths. *Alcohol* 45 2448–2464. 10.1111/acer.14733 34716704 PMC9299043

[B19] Green C. R. Hewitt A. Reynolds J. N. Roane J. Muhajarine N. Mushquash C. (2014). Frequent behavioural challenges in children with fetal alcohol spectrum disorder: A needs-based assessment reported by caregivers and clinicians. *J. Popul. Ther. Clin. Pharmacol.* 21 e405–e420. 25658693

[B20] Gunty A. L. (2020). Rethinking resilience in families of children with autism spectrum disorders. *Couple Fam. Psychol.* 10 87–102. 10.1037/cfp0000155

[B21] Hoffman L. Marquis J. Poston D. Summers J. A. Turnbull A. (2006). Assessing family outcomes: psychometric evaluation of the beach center family quality of life scale. *J. Marr. Fam.* 68 1069–1083. 10.1111/j.1741-3737.2006.00314.x

[B22] Hohlfeld A. Harty M. Engel M. (2018). Parents of children with disabilities: a systematic review of parenting intervention and self-efficacy. *Afri. J. Disabil.* 7:437. 10.4102/ajod.v7i0.437 PMC6244143 30473997

[B23] Joseph J. J. Mela M. Pei J. (2022). Aggressive behaviour and violence in children and adolescents with FASD: a synthesizing review. *Clin. Psychol. Rev.* 94:102155. 10.1016/j.cpr.2022.102155 35397441

[B24] Kempes M. Matthys W. de Vries H. van Engeland H. (2005). Reactive and proactive aggression in children A review of theory, findings and the relevance for child and adolescent psychiatry. *Eur. Child Adoles. Psychiatry* 14 11–19. 10.1007/s00787-005-0432-4 15756511

[B25] Ketelaar M. Bogossian A. Saini M. Visser-Meily A. Lach L. (2017). Assessment of the family environment in pediatric neurodisability: a state-of-the-art review. *Dev. Med. Child Neurol.* 59 259–269. 10.1111/dmcn.13287 27696390

[B26] Nash K. Stevens S. Greenbaum R. Weiner J. Koren G. Rovet J. (2015). Improving executive functioning in children with fetal alcohol spectrum disorders. *Child Neuropsychol.* 21 191–209. 10.1080/09297049.2014.889110 25010354

[B27] Olson D. H. (2011). FACES IV and the circumplex model: validation study. *J. Mar. Fam. Ther.* 37 64–80. 10.1111/j.1752-0606.2009.00175.x 21198689

[B28] Olthuis J. V. McGrath P. J. Cunningham C. E. Boyle M. H. Lingley-Pottie P. Reid G. J. (2018). Distance-delivered parent training for childhood disruptive behavior (Strongest Families™): a randomized controlled trial and economic analysis. *J. Abnor. Child Psychol.* 46 1613–1629. 10.1007/s10802-018-0413-y 29516341

[B29] Pei J. Kapasi A. Kennedy A. E. Joly V. (2019). *Towards healthy outcomes for individuals with fetal alcohol spectrum disorder. Canada FASD research network in collaboration with the University of Alberta*. Edmonton, AB: University of Alberta.

[B30] Peters M. D. J. Godfrey C. M. Khalil H. McInerney P. Parker D. Soares C. B. (2015). Guidance for conducting systematic scoping reviews. *JBI Evidence Implement.* 13 141–146. 10.1097/XEB.0000000000000050 26134548

[B31] Petrenko C. L. M. Demeusy E. M. Alto M. E. (2019). Six-month follow-up of the families on track intervention pilot trial for children with fetal alcohol spectrum disorders and their families. *Alcohol. Clin. Exp. Res.* 43 2242–2254. 10.1111/acer.14180 31408192 PMC6779497

[B32] Petrenko C. L. M. Pandolfino M. E. Robinson L. K. (2017). Findings from the families on track intervention pilot trial for children with fetal alcohol spectrum disorders and their families. *Alcohol. Clin. Exp. Res.* 41 1340–1351. 10.1111/acer.13408 28440861 PMC5534133

[B33] Politte L. C. Fitzpatrick S. E. Erickson C. (2019). *Aggression in autism spectrum Disorder and other Neurodevelopmental Disorders. In Aggression: Clinical Features and Treatment across the lifespan.* Washington, DC: American Psychiatry Association Publishing.

[B34] Popova S. Lange S. Poznyak V. Chudley A. E. Shield K. D. Reynolds J. N. (2019). Population-based prevalence of fetal alcohol spectrum disorder in Canada. *BMC Public Health* 19:845. 10.1186/s12889-019-7213-3 31253131 PMC6599312

[B35] Ramirez J. Andreu J. (2006). Aggression, and some related psychological constructs (anger, hostility, and impulsivity) some comments from a research project. *Neurosci. Biobehav. Rev.* 30 276–291. 10.1016/j.neubiorev.2005.04.015 16081158

[B36] Sanders M. R. Baker S. Turner K. M. T. (2012). A randomized controlled trial evaluating the efficacy of Triple P Online with parents of children with early-onset conduct problems. *Behav. Res. Ther.* 50 675–684. 10.1016/j.brat.2012.07.004 22982082

[B37] Sikora D. Moran E. Orlich F. Hall T. A. Kovacs E. A. Delahaye J. (2013). The relationship between family functioning and behavior problems in children with autism spectrum disorders. *Res. Autism Spect. Dis.* 7 307–315. 10.1016/j.rasd.2012.09.006

[B38] Toole-Anstey C. Keevers L. Townsend M. (2021). A systematic review of child to parent violence interventions. *Trauma Violence Abuse* 24 1157–1171. 10.1177/15248380211053618 34866496

[B39] Tricco A. C. Lillie E. Zarin W. O’Brien K. K. Colquhoun H. Levac D. (2018). PRISMA extension for scoping reviews (PRISMA-SCR): checklist and explanation. *Ann. Internal Med.* 169 467–473. 10.7326/M18-0850 30178033

[B40] Trute B. Hiebert-Murphy D. (2002). Family adjustment to childhood developmental disability: a measure of parent appraisal of family impacts. *J. Pediatric Psychol.* 27 271–280. 10.1093/jpepsy/27.3.271 11909934

[B41] Tsang T. W. Lucas B. R. Olson H. C. Pinto R. Z. Elliott E. J. (2016). Prenatal alcohol exposure, FASD, and child behavior: a meta-analysis. *Pediatrics* 137:e20152542. 10.1542/peds.2015-2542 26908693

[B42] Waltes R. Chiocchetti A. Freitag C. (2016). The neurobiological basis of human aggression: a review on genetic and epigenetic mechanisms. *Am. J. Med. Genet.* 171 650–675. 10.1002/ajmg.b.32388 26494515

[B43] Watson S. L. Coons K. D. Hayes S. A. (2013). Autism spectrum disorder and fetal alcohol spectrum disorder. Part I: a comparison of parenting stress. *J. Intell. Dev. Disabil.* 38 95–104. 10.3109/13668250.2013.788136 23672658

